# Editorial: Critical care applications: bridging high, medium and low-income settings

**DOI:** 10.3389/fmed.2024.1376791

**Published:** 2024-03-01

**Authors:** Luigi Pisani, Wangari Waweru Siika, Madiha Hashmi

**Affiliations:** ^1^Mahidol Oxford Tropical Research Unit (MORU), Bangkok, Thailand; ^2^Department of Precision-Regenerative Medicine and Jonic Area (DiMePRe-J), Section of Anesthesiology and Intensive Care Medicine, University of Bari “Aldo Moro”, Bari, Italy; ^3^Department of Anesthesia, Aga Khan University, Nairobi, Kenya; ^4^Department of Critical Care Medicine, Ziauddin University Hospital, Karachi, Pakistan

**Keywords:** low and middle income countries (LMIC), pneumotacograph, frugal intensive care, protective ventilation, mechanical ventilation

Critical care is an exponentially expanding discipline in low- and middle-income countries (LMICs). As with any other undertaking, the fundamental requirements for the advancement of contemporary and sustainable critical care in LMICs include individuals, processes, and technologies (1). This Research Topic places particular emphasis on processes and technologies, highlighting instances of critical care applications ranging from respiratory monitoring to epidemiological studies.

The study conducted by Farré et al. introduces an innovative, inexpensive method for developing and calibrating low-cost pneumotachographs, specifically designed to measure flow and volume in the context of mechanical ventilation. These devices demonstrated adequate performance under realistic ventilation conditions. Frugal innovation entails the utilization of resource-efficient approaches to technology development and is increasingly being applied in critical care (2–4). Accurate knowledge of the tidal volume administered to patients is crucial to preventing potential harm. While there is evidence suggesting that protective ventilation practices in low- and middle-income countries (LMICs) are comparable to those in high-income countries (HICs) (5, 6), it is essential to underscore the importance of basic practices, such as low tidal volume ventilation.

Protective ventilation extends beyond tidal volume to include the avoidance of both hypoxemia and hyperoxemia in acutely hypoxemic patients. A considerable portion of the existing research originates from high-resource settings and may inadvertently overlook critical contextual factors, as elucidated in the scoping review conducted by Herbst et al.. This review systematically addresses issues such as the potential bias in pulse oximetry measurements, occult hypoxemia, strategies for oxygen conservation, and the lack of data from blood gas analysis resulting in the inability to identify hypercapnic patients. After a comprehensive evaluation of the existing evidence base, guidelines, and ongoing trials, the authors advocate for a target SpO_2_ range between 90 and 94% in a forthcoming large-scale trial involving acutely hypoxemic patients, set to commence in three African countries.

Similar to advances in protective ventilation, various processes are attaining elevated standards in low- and middle-income countries (LMICs). The potential advantages of early mobilization in the intensive care unit (ICU) continue to be debated and scrutinized in randomized trials. In a survey involving over 170 health professionals in Chile, Barros-Poblete et al. reveal a lack of protocols for early mobilization. However, they also highlight a discernible improvement in the skills and infrastructure required for its implementation in ICUs (Barros-Poblete et al.). The gap in early mobilization between LMICs and HICs is closing down, with expert health professionals at the heart of this quality improvement.

While prediction models may be an overused theme in research, the ongoing quest to identify the most useful prediction tool for critically ill patients in low-resource ICUs remains a daily challenge for intensivists in LMICs. In their study, Brotherton et al. from Kenya conducted a comparative analysis of four LMIC-friendly clinical prediction scores for in-hospital mortality in 338 patients admitted to an academic hospital ICU in Kenya. The scores evaluated included the modified early warning score (MEWS), the quick sequential organ failure assessment (qSOFA), the Rwanda Mortality Probability Model (RMPM), and the Tropical Intensive Care Score (TropICS). While no single score demonstrated superiority over the others, the exclusion of TropICS due to excess missing data was notable. Despite the clear limitation of not comparing these scores to more comprehensive models, the study effectively highlights the crucial consideration of balancing predictive model performance with practical feasibility in LMIC settings.

The characteristics of patients with Coronavirus disease 2019 (COVID-19) have been extensively investigated in recent years. Ab Rahman et al. report epidemiological characteristics and outcomes from 19 ICUs in Malaysia. The reported high mortality aligns with findings from other international cohorts (7), further emphasizing the diminishing gap between middle-income Asian countries and their high-income counterparts in terms of COVID-19 outcomes.

While it is imperative to distinguish between low-resource settings and low-income settings (8) and to avoid paternalistic approaches, we maintain the significance of highlighting studies conducted from an LMIC perspective in terms of study design, population, or execution. It is also pivotal to unleash applications from LMICs that exhibit considerable potential to improve the quality of care in high-resource settings.

## Author contributions

LP: Conceptualization, Supervision, Writing – original draft, Writing – review & editing. WS: Writing – original draft, Writing – review & editing. MH: Writing – original draft, Writing – review & editing.
